# Quantum discord of thermal two-photon orbital angular momentum state: mimicking teleportation to transmit an image

**DOI:** 10.1038/s41377-021-00585-8

**Published:** 2021-07-20

**Authors:** Lixiang Chen

**Affiliations:** Department of Physics and Collaborative Innovation Center for Optoelectronic Semiconductors and Efficient Devices, Xiamen University, Xiamen, 361005 China

**Keywords:** Quantum optics, Imaging and sensing

## Abstract

We formulate a density matrix to fully describe two-photon state within a thermal light source in the photon orbital angular momentum (OAM) Hilbert space. We prove the separability, i.e., zero entanglement of the thermal two-photon state. Still, we reveal the hidden quantum correlations in terms of geometric measures of discord. By mimicking the original protocol of quantum teleportation, we demonstrate that the non-zero quantum discord can be utilized to transmit a high-dimensional OAM state at the single-photon level. It is found that albeit the low fidelity of teleportation due to the inherent component of maximally mixed state, the information of all parameters that characterize the original state can still be extracted from the teleported one. Besides, we demonstrate that the multiple repetitions of the protocol, enable the transmission of a complex-amplitude light field, e.g., an optical image, regardless of being accompanied with a featureless background. We also distinguish our scheme of optical image transmission from that of ghost imaging.

## Abstract (web summary)

The formulation of two-photon orbital angular momentum state within a thermal light enables the revelation of its hidden quantumness with non-zero quantum discord and a proposal of teleporting an image.

## Graphical abstract

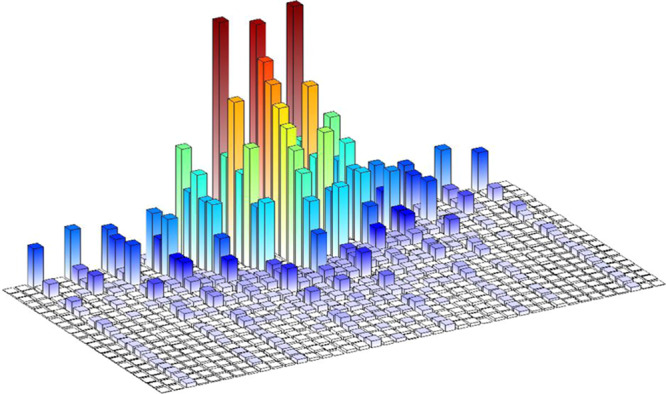

## Introduction

In a seminal paper in 1993^1^, Bennett and coworkers described the quantum protocol of teleportation to transfer any unknown quantum state from one location to another distant one, without physical traveling of the object itself. Its first experimental realization was achieved by Bouwmeester and his colleagues in 1997 using photonic polarization entanglement^2^. Since then, quantum teleportation has been playing an active role in the progress of both quantum information science and technology^3,4^. A lot of research efforts have been triggered to demonstrate numerous variants of teleportation schemes, such as entanglement swapping and quantum repeaters^5^, open-destination teleportation^6^, quantum gate teleportation and quantum computing^7,8^, port-based teleportation^9^, and teleportation of a single photon’s multiple degrees of freedom^10^. The photons are an optimal choice for carrying information in the form of flying qubits and for showcasing nonlocality at a distance such that long-haul experimental teleportation was generally demonstrated with photons^11,12^. Recent progress has been made to realize the teleportation from a transmitter on Earth to a receiver on a satellite, towards a global scale^13^.

In the original scheme of quantum teleportation^1^, two communicating parties, Alice and Bob, must initially share an entangled pair of quantum particles that serves as the quantum channel. For two-level quantum systems, e.g., polarization qubits as in the 1997 experiment^2^, the entangled state generated by parametric down-conversion can be chosen as, $\left| {\Psi} \right\rangle _{A,B}^{( - )} = \frac{1}{{\sqrt {\mathrm{2}} }}\left( {\left| H \right\rangle _A\left| V \right\rangle _B - \left| V \right\rangle _A\left| H \right\rangle _B} \right)$, where *H* and *V* represent the horizontal and vertical polarizations, respectively. In the meantime, Alice is given another third photon *C* whose polarization state $\left| \psi \right\rangle _C$ will be teleported from Alice to Bob. Alice performs a joint Bell measurement on her photons *A* and *C* and obtains one possible outcome of four Bell states, namely, $\left| {\Psi} \right\rangle _{A,C}^{( \pm )} = \frac{1}{{\sqrt {\mathrm{2}} }}\left( {\left| H \right\rangle _A\left| V \right\rangle _C \pm \left| V \right\rangle _A\left| H \right\rangle _C} \right)$ and $\left| {\Phi} \right\rangle _{A,C}^{( \pm )} = \frac{1}{{\sqrt {\mathrm{2}} }}\left( {\left| H \right\rangle _A\left| H \right\rangle _C \pm \left| V \right\rangle _A\left| V \right\rangle _C} \right)$. Then Alice communicates her measurement outcome to Bob via a classical channel. Accordingly, Bob applies a unitary operation, namely the identity operator I or Pauli operators *σ*_*x*_, *σ*_*y*_, *σ*_*z*_ on his photon *B* and recovers the original state. Quantum teleportation has been already generalized to high-dimensional discrete-variable^14,15^ or infinite-dimensional continuous-variable systems^16,17^. Besides, it was demonstrated that some mixed states that contain noisy entanglement, rather than a pure entanglement, can also be exploited as quantum channels for teleportation^18,19^. Moreover, teleportation using only classical channels has been explored and quantified in terms of fidelity bounds^20^. Here, by mimicking the standard protocol of quantum teleportation, we propose a new scheme of using a pseudo-thermal light source to realize the transmission of a high-dimensional orbital angular momentum (OAM) state and even an optical image with quantum discord. Our scheme is based on the formulation of a density matrix on the basis of OAM eigenstates to fully describe two-photon state, which reveals the inherently high-dimensional spatial correlation of non-zero quantum discord. At the single-photon level, the fidelity of the transferred sate is quantified, and for multiple repetitions of our protocol, the distinction of our scheme for optical image transmission is also discussed from ghost imaging.

## Results

### Thermal two-photon state in the OAM space

Our theory is based on the adoption of OAM eigenstates^21,22^, in terms of Laguerre−Gaussian (LG) modes, to formulate the density matrix describing two-photon states within a thermal light source. The quantum theory of the second- and higher-order coherence of light was introduced in 1963 by Glauber^23,24^. Here, we adopt ref. ^25^ to model the thermal radiation as a collection of independent atoms emitting radiation randomly, and its density operator is written as, $\rho _0 = \mathop {\sum}\nolimits_{\{ n_{\mathbf{k}}\} } {P_{\{ n_{\mathbf{k}}\} }\left| {\{ n_{\mathbf{k}}\} } \right\rangle \left\langle {\{ n_{\mathbf{k}}\} } \right|}$, where $P_{\{ n_{\mathbf{k}}\} } = \mathop {\prod}\nolimits_{\mathbf{k}} {P(n_{\mathbf{k}})}$ with *P*(*n*_**k**_) being the probability for *n*_**k**_ photons in the mode **k**, and the symbol {*n*_**k**_} denotes a set of numbers *n*_**k**1_, *n*_**k**2_, *n*_**k**3_,…, etc, of photons excited in very mode. For two-photon case, $n = \mathop {\sum}\nolimits_{\mathbf{k}} {n_{\mathbf{k}}} = 2$, then we can specify $\rho _0 = \mathop {\sum}\nolimits_{{\mathbf{k}},{\mathbf{k}}^\prime } {P\left( {\mathbf{k}} \right)P\left( {{\mathbf{k}}^\prime } \right)\left| {\mathbf{k}} \right\rangle \left| {{\mathbf{k}}^\prime } \right\rangle \left\langle {{\mathbf{k}}^\prime } \right|\left\langle {\mathbf{k}} \right|}$, where $\left| {\mathbf{k}} \right\rangle = \left| {1_{\mathbf{k}}} \right\rangle$ and $\left| {{\mathbf{k}}^\prime } \right\rangle = \left| {1_{{\mathbf{k}}^\prime }} \right\rangle$, denoting only one excited photon in each mode $\left| {\mathbf{k}} \right\rangle$ and $\left| {{\mathbf{k}}^\prime } \right\rangle$, respectively. As the LG modes form an orthogonal and complete computation basis^26^, the light field of any mode $\left| {\mathbf{k}} \right\rangle$ can be expressed in terms of LG modes $\left| {\ell ,p} \right\rangle$, where $\ell$ and *p* are the azimuthal and radial mode indices, respectively. Accordingly, we have

1 $$\rho _0 = \mathop {\sum}\limits_{\ell ,p,\ell ^\prime ,p^\prime } {P_{\ell ,p}P_{\ell ^\prime ,p^\prime }\left| {\ell ,p} \right\rangle \left| {\ell ^\prime ,p^\prime } \right\rangle \left\langle {\ell ^\prime ,p^\prime } \right|\left\langle {\ell ,p} \right|}$$

which indicates that the chaotic source, e.g., thermal light, can be thought of as incoherent statistical mixtures of photon pairs, $\left| {\ell ,p} \right\rangle \left| {\ell ^\prime ,p^\prime } \right\rangle$.

**Fig. 1:**
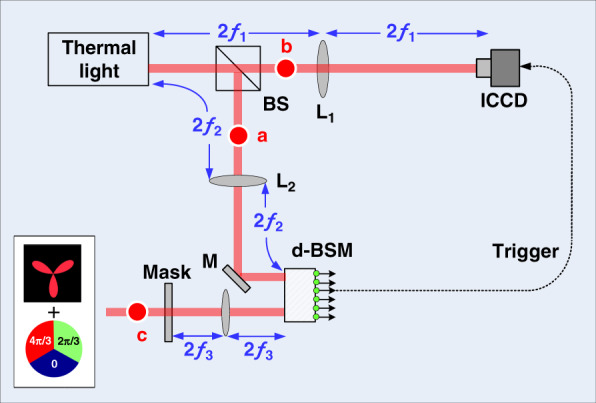
The proposed experimental setup. A thermal light source is utilized to mimick teleportation to transmit a high-dimensional quantum state or an optical image, see the text for details.

In our proposed scheme sketched in Fig. 1, the light derived from a thermal source is directed to a non-polarizing beam splitter (BS) and then is divided into two paths. The interaction with BS changes the two-photon state to,

2 $$\rho _1 = \left| {\Psi} \right\rangle _1\left\langle {\Psi} \right|$$

where

3 $$\left| {\Psi} \right\rangle _1 = \frac{1}{2}\left( {\left| {\ell ,p} \right\rangle _a\left| {\ell ^\prime ,p^\prime } \right\rangle _a + {\mathrm{i}}\left| {\ell ,p} \right\rangle _a\left| {\ell ^\prime ,p^\prime } \right\rangle _b + {\mathrm{i}}\left| {\ell ^\prime ,p^\prime } \right\rangle _a\left| {\ell ,p} \right\rangle _b - \left| {\ell ,p} \right\rangle _b\left| {\ell ^\prime ,p^\prime } \right\rangle _b} \right)$$

Subsequently, we use coincident measurement between photons in two paths, which means that it is just this joint detection that post selects the following two-photon state,

4 $$\rho = \mathop {\sum}\limits_{\ell ,p,\ell ^\prime ,p^\prime } {P_{\ell ,p}P_{\ell ^\prime ,p^\prime }\left| {{\Psi}(\ell ,p;\ell ^\prime ,p^\prime )} \right\rangle _{ab}\left\langle {{\Psi}(\ell ,p;\ell ^\prime ,p^\prime )} \right|}$$

where $\left| {{\Psi}(\ell ,p;\ell ^\prime ,p^\prime )} \right\rangle _{ab} = \left( {\left| {\ell ,p} \right\rangle _a\left| {\ell ^\prime ,p^\prime } \right\rangle _b + \left| {\ell ^\prime ,p^\prime } \right\rangle _a\left| {\ell ,p} \right\rangle _b} \right)/\sqrt 2$. It is found that Eq. (4) can be equivalently written as the following density matrix (see Supplementary Information for details),

5 $$\rho = \rho ^0_{ab} + \left| {\Psi} \right\rangle _{ab}\left\langle {\Psi} \right|$$

where

6 $$\rho ^0_{ab} = \mathop {\sum}\limits_{\ell ,p} {P_{\ell ,p}\left| {\ell ,p} \right\rangle _a\left\langle {\ell ,p} \right|} \mathop {\sum}\limits_{\ell ^\prime ,p^\prime } {P_{\ell ^\prime ,p^\prime }\left| {\ell ^\prime ,p^\prime } \right\rangle _b\left\langle {\ell ^\prime ,p^\prime } \right|}$$

7 $$\left| {\Psi} \right\rangle _{ab} = \mathop {\sum}\limits_{\ell ,p} {P_{\ell ,p}\left| {\ell ,p} \right\rangle _a\left| { - \ell ,p} \right\rangle _b}$$

with subscript, *a* and *b* denote the photon *a* and *b* in two paths, respectively. We show the physically intuitive quantum−mechanical structure of Eq. (5), which describes the two-photon state within a thermal light source: $\rho ^0_{ab}$ of Eq. (6) is merely a diagonal fully separable state, corresponding to the maximally mixed state. In contrast, $\left| {\Psi} \right\rangle _{ab}$ of Eq. (7) is inherently a pure entangled state, which behaves in a very analogous way to the OAM entanglement^27^.

To further derive the specific expression of $P_{\ell ,p}$, we consider the chaotic thermal light source’s cross-spectrum density function. Because of its generality and validity, the Gaussian−Schell model was extensively employed, which can be written as^28^,

8 $$W({\mathbf{r}}_1{\mathbf{,r}}_2) = G_0\exp \left( { - \frac{{r_1^2 + r_2^2}}{{4\sigma _{S}^2}} - \frac{{({\mathbf{r}}_1 - {\mathbf{r}}_2)^2}}{{2\sigma _{g}^2}}} \right)$$

where *G*_0_ is a constant, *σ*_*S*_ is the transverse size, and *σ*_*g*_ is the transverse coherence width of the source. In biphoton case, two-photon amplitude, ${\Phi}({\mathbf{r}}_1{\mathbf{,r}}_2)$, can be expressed as the single-sum expansion based on the Schmidt decomposition, ${\Phi}({\mathbf{r}}_1{\mathbf{,r}}_2) = \mathop {\sum}\nolimits_n {\sqrt {\lambda _n} } u_n({\mathbf{r}}_1)v_n({\mathbf{r}}_2)$, where *u*_*n*_(**r**_1_) and *v*_*n*_(**r**_2_) are Schmidt modes for signal and idler photons, respectively, and *λ*_*n*_ is the eigenvalue^29^. We perform a similar mode decomposition on *W*(**r**_1_, **r**_2_) in the full set of normalized LG modes, i.e., *W*(**r**_1_*,* **r**_2_) = ∑_*ℓ*,*p,ℓ*′,*p*′_*ƒ*_*ℓ*_,_*ℓ*′*,p,p*′_ LG_*ℓ,*__*p*_ (**r**_1_)LG_*ℓ*′,*p*′_(**r**_2_). After some algebra (see Supplementary Information for details), we can find that,

9 $${\it{P}}_{\ell ,{\it{p}}} = \left( {1 - {\mathrm{tan}}^4\frac{\beta }{2}} \right)\left( {{\mathrm{tan}}^2\frac{\beta }{2}} \right)^{|\ell |+2{\it{p}}}$$

where tan *β* = 2*σ*_*S*_/*σ*_*g*_. The main incentive of our present work is to explore the thermal two-photon OAM state of Eq. (5) for the possibility of mimicking teleportation to realize the transmission of a high-dimensional OAM state and even a two-dimensional complex-amplitude optical image, regardless of no prior entanglement.

### Proof of the separability of thermal two-photon OAM state

Based on the concept of robustness of entanglement, we first prove the separability of two-photon state of Eq. (5). Following the procedure presented in ref. ^30^, we know the robustness of entanglement of Eq. (7) $\left( {\rho _Q = \left| {\Psi} \right\rangle _{ab}\left\langle {\Psi} \right|} \right)$ is $R = \left( {\mathop {\sum}\nolimits_{\ell ,p} {P_{\ell ,P}} } \right)^2 - 1$, and the local noise, $\rho_S^ - = \frac{1}{R}\mathop {\sum}\nolimits_{\ell \ne \ell ^\prime {\mathrm{,}}\;p \ne p^\prime } {P_{\ell ,p}P_{\ell ^\prime ,p^\prime }\left| {\ell ,p} \right\rangle _A\left\langle {\ell ,p} \right| \otimes \left| {\ell ^\prime ,p^\prime } \right\rangle _B\left\langle {\ell ^\prime ,p^\prime } \right|}$, which is a separable state by construction. In other words, all entanglement in *ρ*_*Q*_ can be washed out after being mixed with $\rho _S^ -$, and the required minimal amount of $\rho _S^ -$ is *R*. Thus the mixture, $\rho _S^ + = \frac{1}{{1 + R}}\left( {\rho _Q + R\rho _S^ - } \right)$, becomes separable. Besides, by considering the fact that $\rho _S^ - = \frac{1}{R}\left( {\hat \rho _{ab}^0 - \mathop {\sum}\nolimits_{\ell ,p} {P_{\ell ,p}^2} \left| {\ell ,p} \right\rangle _A\left\langle {\ell ,p} \right| \otimes \left| {\ell ,p} \right\rangle _B\left\langle {\ell ,p} \right|} \right)$, we can rewrite Eq. (5) as,

10 $$\rho = \left( {1 + R} \right)\rho _S^ + + \mathop {\sum}\nolimits_{\ell ,p} {P_{\ell ,p}^2\left| {\ell ,p} \right\rangle _A\left\langle {\ell ,p} \right| \otimes \left| {\ell ,p} \right\rangle _B\left\langle {\ell ,p} \right|}$$

which is obviously separable. Thus we have proved that the thermal two-photon source is not entangled per se. However, our theoretical formulation also provides an intuitive picture to reveal the quantum correlations hidden in thermal two-photon OAM state.

### The revelation of the quantumness of thermal two-photon OAM state

When quantum correlation is mentioned, we might be immediately tempted to think of entanglement. But it deserves our attention that entanglement is not the only aspect of quantum correlations, namely, some quantum systems even without entanglement are capable of possessing correlations that cannot be simulated by classical physics^31^. In the OAM Hilbert space, we have formulated the density operator of Eq. (5) to fully describe the thermal two-photon source. It is crucial to distinguish quantum correlations beyond entanglement. In parallel with the entanglement measure used for entanglement vs separability criteria, discord was proposed as a figure of merit for characterizing the nonclassical resource in the quantum vs classical scenario^32,33^ It is defined as the discrepancy between quantum mutual information (total correlation) and classical correlation in a bipartite system. However, the evaluation of quantum discord requires considerable numerical minimization. From a geometric perspective, a distance-based notion of discord^34^, was introduced instead. For an arbitrary state *ρ*, this geometric discord is defined as $D\left( \rho \right) = \mathop {{\min }}\limits_{\chi \in {\Omega}_0} \left\| {\rho - \chi } \right\|^2$, where Ω_0_ denotes the set of zero-discord states, and $\left\| {\rho - \chi } \right\|^2 = {\mathrm{tr}}\left( {\rho - \chi } \right)^2$ is the square norm in the Hilbert-Schmidt space. Progress in computing *D*(*ρ*) has been made by showing that^35^,

11 $$D\left( \rho \right) = \mathop {{\min }}\limits_{\left| {\eta _k^B} \right\rangle } \left[ {{\mathrm{tr}}\left( {\rho ^2} \right) - \mathop {\sum}\limits_k {\left\langle {\eta _k^B} \right|\rho \left| {\eta _k^B} \right\rangle ^2} } \right]$$

where the minimization is taken over all local bases $\left| {\eta _k^B} \right\rangle$ on photons in path B.

After a lengthy algebra based on Eq. (11), we can obtain the analytic geometric discord for Eq. (5) as (see Supplementary Information),

12 $$D\left( \rho \right) = \frac{{\left( {\mathop {\sum}\nolimits_{\ell ,p} {P_{\ell ,p}^2} } \right)^2 - \mathop {\sum}\nolimits_{\ell ,p} {P_{\ell ,p}^4} }}{{\left( {\mathop {\sum}\nolimits_{\ell ,p} {P_{\ell ,p}^2} + \left( {\mathop {\sum}\nolimits_{\ell ,p} {P_{\ell ,p}^{}} } \right)^2} \right)^2}}$$

**Fig. 2:**
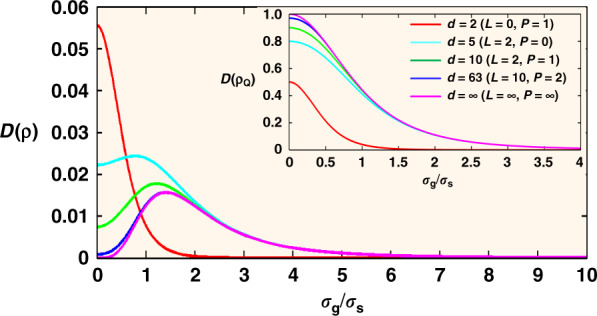
Geometric measure of discord with various dimensions. *D*(*ρ*) describes the thermal two-photon state of Eq. (5), while the inset *D*(*ρ*_*Q*_) corresponds to the pure entangled state of Eq. (7).

We have plotted *D*(*ρ*) in Fig. 2 in different OAM subspaces, whose dimension is specified by $d = \left( {2L + 1} \right)\left( {P + 1} \right)$, with $\ell$ ranging from −*L* to *L* and *p* from 0 to *P*. One can see that, regardless of zero entanglement, the quantum discord is surprisingly non-zero, and therefore revealing the quantumness of correlations hidden in the thermal two-photon state. We also plot the geometric discord *D*(*ρ*_*Q*_) for the pure entangled state of Eq. (7) within the same subspaces. Two extreme cases, *σ*_*g*_ = ∞ and *σ*_*g*_ = 0, deserve our special attention. For *σ*_*g*_ = ∞, the source is completely coherent, and none of the quantum correlations can be extracted from either the thermal state or the entangled state, as $D( \rho ) = D({\rho}_Q) = 0$. For *L* = *P* = ∞, we can also obtain, $D_\infty \left( \rho \right) = 1/\left( {\sigma _{\mathrm{g}}/\sigma _{\mathrm{s}} + 2\sigma _{\mathrm{s}}/\sigma _{\mathrm{g}}} \right)^4$, which is shown by the purple curves in Fig. 2. The other extreme is the case of the completely incoherent light source, where *ρ*_*Q*_ is maximally entangled and $P_{\ell ,p}$ becomes a constant. For comparison, if we also apply the geometric discord to the initial state of Eq. (1), a straightforward analysis finds *D*(*ρ*_0_) = 0. Thus we are allowed to conclude that it is just the function of BS and the post-selection of joint detection that extract the nonclassical correlations. As we will demonstrate below, we can still utilize such a non-entangled yet non-classical thermal two-photon state to realize the transmission of a high-dimensional OAM state at the single-photon level and that of an optical image with multiple repetitions of our protocol.

### Mimicking teleportation to transmit the high-dimensional OAM state of a single photon

As shown in Fig. 1, we use an additional mirror (M) in the path of photon b to flip the OAM value. For simplicity and without loss of generality, we consider a completely incoherent source with *σ*_*g*_ = 0, which leads to $\tan \frac{\beta }{2} = 1$ and indicates that $P_{\ell ,p}$ becomes a constant, as it is independent of $\ell$ or *p*. If we further consider a subspace with the radial index *p* ranging from 0 to *P* and the azimuthal index $\ell$ from −*L* to +*L*, we have $d = \left( {P + 1} \right)\left( {2L + 1} \right)$ denotes the dimension. Besides, for convenience, we reindex all the eigenstates of LG modes by assigning $\left| j \right\rangle = \left| {\ell ,p} \right\rangle$, with $j = \ell + \left( {2L + 1} \right)p + L$ ranging from *j* = 0 to *d* − 1. As a result, we can rewrite Eqs. (6) and (7) as,

13 $$\rho ^0_{ab} = \frac{1}{d}\mathop {\sum}\limits_j {\left| j \right\rangle _a\left\langle j \right|} {\mathrm{ }}\mathop {\sum}\limits_{j^\prime } {\left| {j^\prime } \right\rangle _b\left\langle {j^\prime } \right|}$$

14 $$\left| {\Psi} \right\rangle _{ab} = \frac{1}{{\sqrt d }}\mathop {\sum}\limits_j {\left| j \right\rangle _a} \left| j \right\rangle _b$$

Here we explore the above quantum correlations hidden in the thermal two-photon state to serve as a quantum bridge to accomplish the transmission of a high-dimensional OAM state for a single photon, akin to the standard teleportation protocol. In Fig. 1, the photons *a* and *b* in two paths are spatially separated. Assume Alice holds photon *a* while Bob possesses photon *b*. To mimic the teleportation, Alice has a third photon *c* at hand, which is encoded with the high-dimensional OAM state, mathematically corresponding to the complex light field of an optical image, *ψ*(**r**). This is realized by passing the photon *c* in an initial fundamental Gaussian mode through an encoded holographic mask. According to the concept of digital spiral imaging^26,36^, we can rewrite the complex light field in terms of LG modes as, $\left| \psi \right\rangle _c = \mathop {\sum}\nolimits_j {A_j} \left| j \right\rangle _c$, where $A_j = A_{\ell ,p} = {\int} {\left[ {{\mathrm{LG}}_{\ell, p} ({\mathbf{r}})} \right]^ \ast \psi ({\mathbf{r}}){\mathrm{d}}{\mathbf{r}}}$. In our proposed experiment, we use a series of imaging lenses to image the plane of a chaotic thermal light source to the plane of an ICCD camera (via 4*f*_1_ for photon *b*) and to the plane of a high-dimensional Bell state measurement device (d-BSM) (via 4*f*_2_ for photon *a*), respectively. Meanwhile, another single imaging lens (4*f*_3_) is used to image the mask onto the d-BSM as well in the path of photon *c*. Then the wavefunction of the whole system can be written as, $\rho _{abc} = \left( { \rho _{ab}^0 + \left| {\Psi} \right\rangle _{ab}\left\langle {\Psi} \right|} \right)\left( {\left| \psi \right\rangle _c\left\langle \psi \right|} \right)$. Here the d-BSM realizes the function of projecting photons *a* and *c* onto the generalized high-dimensional Bell states in the Hilbert space spanned by the LG modes. Then, we are allowed to rewrite the whole state $\rho_{abc}$, in terms of the generalized Bell states of photons *a* and *c*, as

15 $$\rho _{abc} = \mathop {\sum}\limits_{m,n} {\left( {\frac{1}{d}\mathop {\sum}\limits_j {\left| j \right\rangle _b} \left\langle j \right| + \left| \varphi \right\rangle _b\left\langle \varphi \right|} \right)} \left| {\psi ^{mn}} \right\rangle _{ac}\left\langle {\psi ^{mn}} \right|$$

where $\left| {\psi ^{m,n}} \right\rangle _{ac} = \frac{1}{{\sqrt d }}\mathop {\sum}\nolimits_j {{\mathrm{e}}^{{\mathrm{i}}2\pi jm/d}} \left| j \right\rangle _a\left| {\left( {j + n} \right){\mathrm{mode}} \ {d}} \right\rangle _c$ are two-photon generalized Bell states, and $\left| \varphi \right\rangle _b = U_{mn}^ + \left| \psi \right\rangle _b$ with $U_{mn} = \mathop {\sum}\nolimits_k {{\mathrm{e}}^{{\mathrm{i}}2\pi km/d}} \left| k \right\rangle \left\langle {\left( {k + n} \right){\mathrm{mode}} \ {d}} \right|$ being a unitary matrix. Equation (15) forms the key result of the present work, which suggests the possibility of using a thermal light source to mimic the teleportation protocol for realizing the transmission of a high-dimensional OAM state of a single photon and even a complex-amplitude optical image. After performing the generalized Bell state measurement on photons *a* and *c*, Alice gets one possible result out of *d*^2^ outcomes, *k* = (*m*,*n*), where $m,n = 0,1,2,...,d - 1$. Then Alice communicates classically her measurement result *k* = (*mn*) with Bob. Accordingly, Bob applies the conditional unitary matrix *U*_*mn*_ to the photon *b*, namely

16 $$\rho _b = U_{mn}\frac{1}{2}\left( {\frac{1}{d}\mathop {\sum}\limits_j {\left| j \right\rangle _b} \left\langle j \right| + \left| \varphi \right\rangle _b\left\langle \varphi \right|} \right)U_{mn}^ + = \frac{1}{2}\left( {\frac{1}{d}\mathop {\sum}\limits_j {\left| j \right\rangle _b} \left\langle j \right| + \left| \psi \right\rangle _b\left\langle \psi \right|} \right)$$

This suggests that Bob can retrieve an output state of photon *b*, which is just a mixture of an exact replica of the original state of a photon *a*, $\left| \psi \right\rangle _a$, and a maximally mixed state, $\frac{1}{d}\mathop {\sum}\nolimits_j {\left| j \right\rangle } \left\langle j \right|$.

## Discussion

### Our protocol at the single-photon level

**Fig. 3:**
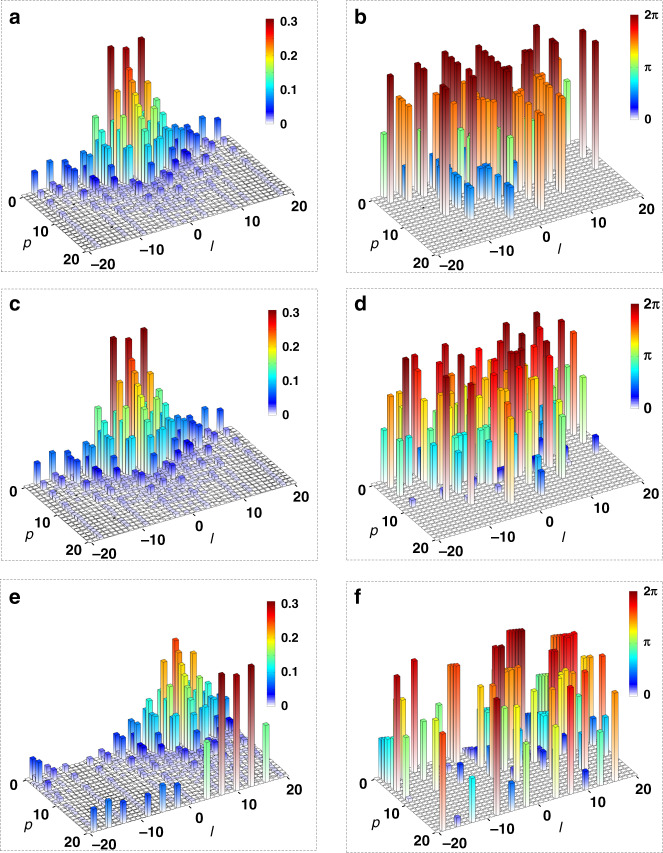
The simulation results of transmitting a high-dimensional state at the single-photon level. **a**, **b** The correct result of $\left| \psi \right\rangle _b$, conditional on $\left| {\psi ^{0,0}} \right\rangle _{ac}$. **c**, **d** The incorrect result of $U_{2,0}^ + \left| \psi \right\rangle _b$, conditional on $\left| {\psi ^{2,0}} \right\rangle _{ac}$; **e**, **f** the incorrect result of $U_{10,10}^ + \left| \psi \right\rangle _b$, conditional on $\left| {\psi ^{10,10}} \right\rangle _{ac}$. Left and right columns show the spectra, $a_{\ell ,p}$ and $\phi _{\ell ,p}$, respectively, while the trivial background of a maximally mixed state $\frac{1}{d}\mathop {\sum}\nolimits_j {\left| j \right\rangle } \left\langle j \right|$ is not shown

We assume that the state encoded by photon *c* is represented by a high-dimensional vector, $|\psi \rangle_c = \mathop {\sum}\nolimits_j A_j \left| j \right\rangle_c = \mathop {\sum}\nolimits_{\ell,p} A_{\ell,p} \left|\ell,p\right\rangle_c$, which can be equivalently characterized by two spectra, $a_{\ell ,p} = \left| {A_{\ell ,p}} \right|$ and $\phi _{\ell ,p} = \arg (A_{\ell ,p})$, respectively. It is noted that in classical optics, such a high-dimensional state just corresponds to a complex-amplitude optical image, namely, $\psi \left( {\mathbf{r}} \right) = \mathop {\sum}\nolimits_{\ell ,p} {A_{\ell ,p}} {\mathrm{LG}}_{\ell ,p}\left( {\mathbf{r}} \right)$. The calculation of $a_{\ell ,p}$ and $\phi _{\ell ,p}$ for a Clover image is shown in the section of “Materials and methods”. From Eq. (15), without loss of generality, assume the d-BSM results at Alice’s side are $k = (m,n) = (0,0)$, (2,0) and (10,10), namely, $\left| {\psi ^{0,0}} \right\rangle _{ac}$, $\left| {\psi ^{2,0}} \right\rangle _{ac}$, and $\left| {\psi ^{10,10}} \right\rangle _{ac}$, respectively. Accordingly, photon *b* will be projected onto the state of $U_{0,0}^ + \left| \psi \right\rangle _b$, $U_{2,0}^ + \left| \psi \right\rangle _b$, and $U_{10,10}^ + \left| \psi \right\rangle _b$. For an easy visualization, we illustrate in Fig. 3 the spectra $a_{\ell ,p}$ and $\phi _{\ell ,p}$ of these states, in which the trivial background of a maximally mixed state $\frac{1}{d}\mathop {\sum}\nolimits_j {\left| j \right\rangle _b\left\langle j \right|}$ is omitted. In Fig. 3a, b, as $U_{00}^ + = I$, photon *b* just acquires the original state of photon *c*, accompanying with the trivial background. In contrast, conditional on $\left| {\psi ^{2,0}} \right\rangle _{ac}$ and $\left| {\psi ^{10,10}} \right\rangle _{ac}$ and without performing the desired unitary operations, we can only obtain the incorrect high-dimensional state, $U_{2,0}^ + \left| \psi \right\rangle _b$, and $U_{10,10}^ + \left| \psi \right\rangle _b$, respectively. According to $U_{mn} = \mathop {\sum}\nolimits_k {{\mathrm{e}}^{{\mathrm{i}}2\pi km/d}} \left| k \right\rangle \left\langle {(k + n) \ {\mathrm{mode}} \ {d}} \right|$, we have $U_{mn}^ + = \mathop {\sum}\nolimits_k {{\mathrm{e}}^{ - {\mathrm{i}}2\pi km/d}} \left| {(k + n) \ {\mathrm{mode}} \ {d}} \right\rangle \left\langle k \right|$. Therefore, we have $U_{2,0}^ + = \mathop {\sum}\nolimits_k {{\mathrm{e}}^{ - {\mathrm{i}}4\pi k/d}} \left| k \right\rangle \left\langle k \right|$ such that the spectrum of $a_{\ell ,p}$ remains unchanged while each phase $\phi _{\ell ,p}$ is individually shifted by an amount of ${\Delta}\phi = - 4\pi k/d$, see Fig. 3c, d. While for $U_{10,10}^ + = \mathop {\sum}\nolimits_k {{\mathrm{e}}^{ - {\mathrm{i}}20\pi k/d}} \left| {k + 10} \right\rangle \left\langle k \right|$, as shown by Fig. 3e, f, we can see that both spectra of $a_{\ell ,p}$ and $\phi _{\ell ,p}$ are changed. In other words, the two latter cases without desired unitary operations can only lead to the incorrect transferred states.

As well known, quantum teleportation is distinct in that it allows a single copy of a state to be teleported with unity fidelity. Therefore, it is crucial to investigate the fidelity of the transferred state in our protocol, which can be written as,

17 $$F = {\mathrm{Tr}}\left( {\rho _b\left| \psi \right\rangle _b\left\langle \psi \right|} \right) = \frac{1}{2}\left( {\frac{1}{d}\mathop {\sum}\limits_j {\left| j \right\rangle _b} \left\langle j \right| + \left| \psi \right\rangle _b\left\langle \psi \right|} \right)\left| \psi \right\rangle _b\left\langle \psi \right| = \frac{1}{2} + \frac{1}{{2d}}$$

The particular case of *d* = 2 leads to *F* = 3/4, which agrees to the optimal fidelity achievable with a two spin-1/2 Werner stat^37^. However, high dimensions are essential for encoding an optical image. From Eq. (17), we find that the fidelity decreases inversely as the dimensionality increases, approaching to the limit of *F* = 1/2 for $d \to \infty$, due to the inherent existence of the background maximally mixed state of $\frac{1}{d}\mathop {\sum}\nolimits_j {\left| j \right\rangle } \left\langle j \right|$ in Eq. (16). In an actual experiment, the background can only be subtracted by sending multiple copies of the same state, which changes the resources used in the scheme since Alice performing the Bell-state measurement needs to communicate *d* bits to Bob for each repetition of the protocol, corresponding to one of the *d* possible outcomes of the Bell-state measurement. This classical communication itself may exceed the number of bits used to describe the image. However, if the density matrix of Eq. (16) could be reconstructed, e.g., via quantum state tomography, one can see that the information about all the parameters of the original state, $a_{\ell ,p}$ and $\phi _{\ell ,p}$, could still be fully known, Albeit a low fidelity of teleportation.

### Our protocol with multiple repetitions

**Fig. 4:**
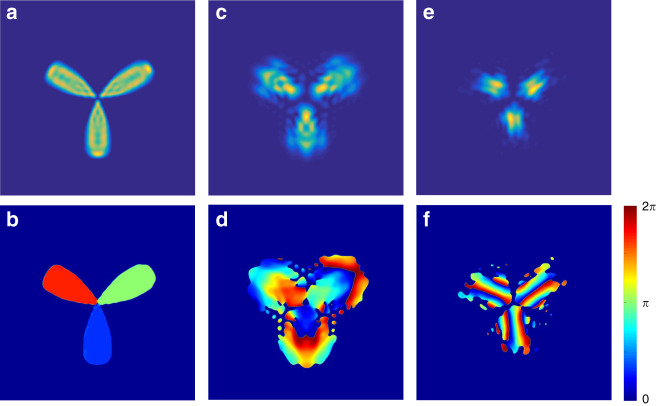
The multiple repetitions of our protocol for transmitting a complex-amplitude Clover image. **a**, **b** The intensity and phase profiles recorded by the ICCD camera with the d-BSM results of $\left| {\psi ^{0,0}} \right\rangle _{ac}$ as the trigger signal **c**, **d** $\left| {\psi ^{2,0}} \right\rangle _{ac}$; **e**, **f** $\left| {\psi ^{10,10}} \right\rangle _{ac}$. Note that the featureless background is not shown trivially.

Reconstruction of an optical image requires multiple single-photon events. Thus it is also interesting to consider the multiple repetitions of our protocol. As mentioned above, the high-dimensional state just corresponds to a complex-amplitude optical image of a Clover, see the inset of Fig. 1. If we post-select the generalized d-BSM result of $\left| {\psi ^{0,0}} \right\rangle _{ac}$, we can record the single photons to reconstruct the Clover image with the ICCD camera. Then we can plot the intensity and phase profiles of the Clover image in Fig. 4a, b, respectively, which corresponds to the high-dimensional state characterized by Fig. 3a, b. Note that the background is trivially omitted. It can be seen that the reconstruction of the pure Clover image is ensured by the second term of Eqs. (15) or (16), which when viewed separately is due to the spatial correlations established by the entangled state of Eq. (7) or Eq. (14). This is just physically equivalent to using a quantum source of biphoton pure entangled state. In contrast, when the ICCD camera is triggered by another d-BSM results, e.g, $\left| {\psi ^{2,0}} \right\rangle _{ac}$ and $\left| {\psi ^{10,10}} \right\rangle _{ac}$, we could only record a fuzzy image, see Fig. 4c–f, respectively, as a result of the incorrect transmissions of the high-dimensional states, see Fig. 3c–f, respectively. In principle, one should perform a desired unitary operations for each repetition in order to recover the correct image.

### Comparison with ghost imaging

As it is known, another well-established application of thermal light source, is the technique of ghost imaging. The ghost imaging could be traced back to the experiment conducted in 1995 that exploited the nonlocal correlations of entangled photon pairs created by parametric down-conversion^38^. However, it was demonstrated that a coincidence image can also be obtained with thermal light source^39–42^, thus raising a question whether entanglement was truly necessary for ghost imaging^43–45^. Several efforts have contributed to providing some insightful understanding on this historic argument^46–48^. Here our scheme uses thermal light, and the recorded images in both two cases are embedded in a featureless background. However, we distinguish our scheme of image teleportation from that of ghost imaging. In ghost imaging, only a pair of photons are employed; one photon in the pair illuminates the object and is collected by a bucket detector while the other photon is utilized to record the images with a spatially resolved detector, e.g., an intensive CCD camera. In contrast, here we employ three photons; two photons (*a* and *b*) in a pair derived from the thermal light source serve as the channel for teleportation, while the third photon (*c*) is directed to illuminate the object and acquires the image information to be teleported. We then follow the standard procedure of teleportation to perform the generalized Bell state measurements (d-BSM) on photons *a* and *c*, and use photon b to record the teleported images with the ICCD camera nonlocally. Besides, it is shown that the correct image could be obtained only after the desired unitary transformation, conditional on the d-BSM results of photons *a* and *c*.

Besides, the formulation of thermal two-photon OAM state, $\rho=\rho_Q+\rho ^0_{ab}$, offers an intuitive picture of two-photon quantum interference. If we look *ρ*_*Q*_ separately, it just accounts for the existence two-photon OAM interference. In contrast, if we look *ρ* wholly, the interference of *ρ*_*Q*_ has been already canceled out by $\rho^0_{ab}$, thus leading to the inexistence of two-photon interference. In this regard, our work can also shed new light on the historical debate as to whether the origin of ghost imaging is quantum-mechanical or classical.

In summary, we have formulated a density matrix on the basis of OAM eigenmodes to fully describe the thermal two-photon state and reveal the quantumness with non-zero discord. We also demonstrate that albeit a low teleportation fidelity, such a thermal two-photon state can be explored for realizing the transmission of a high-dimensional OAM state at the single-photon level and that of an optical image with multiple repetitions of our protocol. This is obviously distinguished from the traditional method of using a prior entangled source. However, as in the case of using entanglement, the practical implementation of our scheme is hindered by the high-dimensional Bell state measurement (d-BSM). For our demonstration, it requires the manipulation of two-photon *d*-level states encoded by LG modes with both azimuthal and radial indices, which remains more challenging with the current experimental technique. In the future, our work can also call for further studies of using thermal multi-photon state to demonstrate some new quantum information tasks, such as remote state preparation^49^ and novel imaging with undetected photons^50^.

## Materials and methods

### The calculation of both intensity and phase spiral spectra for the Clover image

In Fig. 1, the Clover object together with the single-mode fiber and the single-photon detector (not shown) inversely defines the state of photon c, $\left| \psi \right\rangle _c = \mathop {\sum}\nolimits_{\ell ,p} {A_{\ell ,p}\left| {\ell ,p} \right\rangle _c}$, where $A_{\ell ,p} = {\int} {\left( {{\mathrm{LG}}_{\ell, p} ({\mathbf{r}})} \right)^ \ast \psi ({\mathbf{r}}){\mathrm{d}}{\mathbf{r}}}$ denotes the overlap probability amplitude and is related to digital spiral imaging of the Clover object. Accordingly, we present in Fig. 3a, b the intensity spectrum, $a_{\ell ,p}$, and the phase spectrum, $\phi _{\ell ,p}$. Due to the three-fold rotational symmetry of the Clover considered here, one can see that only OAM components of $\ell = 0,\; \pm {\mathrm{3,}}\; \pm {\mathrm{6}}$, dominate in the spiral spectrum. Besides, as the adopted beam waist of the LG modes *w* = 0.4469 mm is comparable to the radius of the Clover object *R* = 1 mm, only those lower-order LG modes with a small *p* index contribute together to form the Clover object.

## Supplementary information

Supplementary information

## References

[1] Bennett CH (1993). Teleporting an unknown quantum state via dual classical and Einstein−Podolsky−Rosen channels. Phys. Rev. Lett..

[2] Bouwmeester D (1997). Experimental quantum teleportation. Nature.

[3] Pan JW (2012). Multiphoton entanglement and interferometry. Rev. Mod. Phys..

[4] Pirandola S (2015). Advances in quantum teleportation. Nat. Photonics.

[5] Żukowski M (1993). “Event-ready-detectors” Bell experiment via entanglement swapping. Phys. Rev. Lett..

[6] Zhao Z (2004). Experimental demonstration of five-photon entanglement and open-destination teleportation. Nature.

[7] Gottesman D, Chuang IL (1999). Demonstrating the viability of universal quantum computation using teleportation and single-qubit operations. Nature.

[8] Huang YF (2004). Experimental teleportation of a quantum controlled-NOT gate. Phys. Rev. Lett..

[9] Ishizaka S, Hiroshima T (2008). Asymptotic teleportation scheme as a universal programmable quantum processor. Phys. Rev. Lett..

[10] Wang XL (2015). Quantum teleportation of multiple degrees of freedom of a single photon. Nature.

[11] Gisin N, Thew R (2007). Quantum communication. Nat. Photonics.

[12] Northup TE, Blatt R (2014). Quantum information transfer using photons. Nat. Photonics.

[13] Ren JG (2017). Ground-to-satellite quantum teleportation. Nature.

[14] Werner RF (2001). All teleportation and dense coding schemes. J. Phys. A: Math. Gen..

[15] Goyal SK (2014). Qudit-teleportation for photons with linear optics. Sci. Rep..

[16] Braunstein SL, Kimble HJ (1998). Teleportation of continuous quantum variables. Phys. Rev. Lett..

[17] Furusawa A (1998). Unconditional quantum teleportation. Science.

[18] Horodecki M, Horodecki P, Horodecki R (1999). General teleportation channel, singlet fraction, and quasidistillation. Phys. Rev. A.

[19] Verstraete F, Verschelde H (2003). Optimal teleportation with a mixed state of two qubits. Phys. Rev. Lett..

[20] Henderson L, Hardy L, Vedral V (2000). Two-state teleportation. Phys. Rev. A.

[21] Padgett M, Courtial J, Allen L (2004). Light’s orbital angular momentum. Phys. Today.

[22] Molina-Terriza G, Torres JP, Torner L (2007). Twisted photons. Nat. Phys..

[23] Glauber RJ (1963). Photon correlations. Phys. Rev. Lett..

[24] Glauber RJ (1963). Coherent and incoherent states of the radiation field. Phys. Rev..

[25] Loudon, R. *The Quantum Theory of Light* 3rd edn, (Oxford University Press, 2000).

[26] Torner L, Torres JP, Carrasco S (2005). Digital spiral imaging. Opt. Express.

[27] Mair A (2001). Entanglement of the orbital angular momentum states of photons. Nature.

[28] Foley JT, Zubairy MS (1978). The directionality of Gaussian Schell-model beams. Opt. Commun..

[29] Law CK, Eberly JH (2004). Analysis and interpretation of high transverse entanglement in optical parametric down conversion. Phys. Rev. Lett..

[30] Vidal G, Tarrach R (1999). Robustness of entanglement. Phys. Rev. A.

[31] Modi K (2012). The classical-quantum boundary for correlations: discord and related measures. Rev. Mod. Phys..

[32] Ollivier H, Zurek WH (2001). Quantum discord: a measure of the quantumness of correlations. Phys. Rev. Lett..

[33] Henderson L, Vedral V (2001). Classical, quantum and total correlations. J. Phys. A: Math. Gen..

[34] Dakić B, Vedral V, Brukner Č (2010). Necessary and sufficient condition for nonzero quantum discord. Phys. Rev. Lett..

[35] Luo SL, Fu SS (2010). Geometric measure of quantum discord. Phys. Rev. A.

[36] Chen LX, Lei JJ, Romero J (2014). Quantum digital spiral imaging. Light.: Sci. Appl..

[37] Popescu S (1994). Bell’s inequalities versus teleportation: what is nonlocality?. Phys. Rev. Lett..

[38] Pittman TB (1995). Optical imaging by means of two-photon quantum entanglement. Phys. Rev. A.

[39] Cheng J, Han SS (2004). Incoherent coincidence imaging and its applicability in X-ray diffraction. Phys. Rev. Lett..

[40] Gatti A (2004). Ghost imaging with thermal light: comparing entanglement and classical correlation. Phys. Rev. Lett..

[41] Valencia A (2005). Two-photon imaging with thermal light. Phys. Rev. Lett..

[42] Ferri F (2005). High-resolution ghost image and ghost diffraction experiments with thermal light. Phys. Rev. Lett..

[43] Scarcelli G, Berardi V, Shih Y (2006). Can two-photon correlation of chaotic light be considered as correlation of intensity fluctuations?. Phys. Rev. Lett..

[44] Gatti A (2007). Comment on “can two-photon correlation of chaotic light be considered as correlation of intensity fluctuations?”. Phys. Rev. Lett..

[45] Scarcelli G, Berardi V, Shih YH (2007). Scarcelli, Berardi, and Shih reply. Phys. Rev. Lett..

[46] Zhang MH (2007). Sub-wavelength Fourier-transform imaging of a pure-phase object with thermal light. Phys. Lett. A.

[47] Meyers R, Deacon KS, Shih Y (2008). Ghost-imaging experiment by measuring reflected photons. Phys. Rev. A.

[48] Erkmen BI, Shapiro JH (2008). Unified theory of ghost imaging with Gaussian-state light. Phys. Rev. A.

[49] Dakić B (2012). Quantum discord as resource for remote state preparation. Nat. Phys..

[50] Lemos GB (2014). Quantum imaging with undetected photons. Nature.

