# Supplementary material for: Quantum discord of thermal two-photon orbital angular momentum state: mimicking teleportation to transmit an image
Source: Light Sci Appl. 2021 Jul 20;10:148. doi: 10.1038/s41377-021-00585-8 (PMC8292362; doi:10.1038/s41377-021-00585-8)
Supplement: Supplementary file 1 — Supplementary information [file 41377_2021_585_MOESM1_ESM.docx]

Supplementary Information for

Quantum discord of thermal two-photon orbital angular momentum state: Mimicking teleportation to transmit an image

Lixiang Chen*

*Department of Physics and Collaborative Innovation Center for Optoelectronic Semiconductors and Efficient Devices, Xiamen University, Xiamen 361005, China*

***[*chenlx@xmu.edu.cn*](mailto:*chenlx@xmu.edu.cn)

**I. The derivation of Eq. (5)**

The LG mode is a natural choice to describe twisted photons carrying orbital angular momentum (OAM). In the cylindrical coordinates , the normalized form of a Laguerre-Gaussian (LG) mode is mathematically given by [1],

(S1)

The beam radius at $z$ is specified by $\omega(z)=\omega_{0}\sqrt{1+z/z_{R}}$ with $z_{R}=\pi\omega_{0}^{2}/\lambda$ being the Rayleigh range and $\omega_{0}$ being the beam waist radius. The arctangent term is the Gouy phase, and the helical phase term is associated with OAM per photon. Let us start with the thermal two-photon state that is represented in the basis of LG modes as,

, (S2)

where are defined at the beam waist $z=0$. This indicates that the thermal sources can be thought of as incoherent statistical mixtures of photon pairs, , all independent from each other. For the standard two-arm configuration of Fig. (1), however, if the effects of the beam splitter and the joint detection are combined to take into account, then, surprisingly enough, we are able to distill some quantum correlations from Eq. (S2). The effect of a spatially symmetric beam splitter (BS) on an incoming photon can be represented by a transformation matrix [2],

$\mathbf{B}=\frac{1}{\sqrt{2}}\left[\begin{matrix} \begin{matrix} i & 1 \end{matrix} \\ \begin{matrix} 1 & i \end{matrix} \end{matrix}\right]$, (S3)

which indicates that each mode acquires a phase shift of $\pi/2$ due to reflection. Additionally, the reflection flips the transverse profile of the incident mode such that the sign of OAM is reversed, namely, , which is subsequently compensated by a reflective prism in the image arm. Thus after BS and prism, we have the state,

$\hat{\rho}_{1}=|\Psi\rangle_{1}\langle\Psi|$, (S4)

where

. (S5)

We use coincident measurement between photons in two paths, which means that it is just this joint detection that postselects the following two-photon state,

. (S6)

where . In particular, if or $p\neq p'$, then can be referred to as a spatial entanglement that resides in the Hilbert subspace spanned by the OAM base of , for photon a and , for photon b. In other words, can be equivalent to a maximally entangled Bell state [3]. However, the density matrix of Eq. (S6), as a statistical mixture of , does not always indicate the existence of quantum entanglement, depending on whether they can be rearranged as a convex sum of different product states. However, to reveal the possible quantum aspect that is concealed by Eq. (S6), it is tempting and illuminating for us to express it as a sum of two contributions,

$\hat{\rho}=\hat{\rho}_{ab}^{0}+\hat{\rho}_{Q}$, (S7)

where

, (S8)

, (S9)

It is noted that for the LG modes at beam waist $z=0$, we have the relation, . Then we are allowed to rewrite Eq. (S9) as,

. (S10)

By considering Eq. (S10), we can rewrite Eq. (S7) as,

$\rho=\hat{\rho}_{ab}^{0}+|\Psi\rangle_{ab}\langle\Psi|$, (S11)

where

. (S12)

Thus we have derived Eqs. (5) to (7).

**II. The derivation of Eq. (9)**

The Gaussian-Schell model of Eq. (8) is generally used to describe the partially coherent radiation sources [4], which can be rewritten as,

(S13)

We perform the Schmidt decomposition in the basis of LG modes at beam waist $z=0$, namely,

, (S14)

where denotes the probability amplitude to find one photon in the mode while the other photon in the mode. As the LG modes form a complete and orthogonal basis in the OAM Hilbert space, we know that,

. (S15)

By utilizing the Anger-Jacobi identity [5], , we obtain from Eqs. (S13) and (S14) the selection rule for index, namely . Besides, it is tempting to perform the first radial integral for $r_{1}$ in Eq. (S15) analytically by virtue of the formula

. (S16)

Afterwards, the integral for $\rho_{2}$ can also be done analytically just by setting both the beam waists of and modes as $w_{A}=w_{B}=2\sigma_{S}\sqrt{\cos\beta}$, with $\tan\beta=2\sigma_{S}/\sigma_{g}$. Under this special condition, we have the similar selection rule for $p$ index, namely, $p'=p$. Subsequently, by utilizing the orthogonality of LG modes, namely , we are able to finally obtain Eq. (9).

**III. The derivation of Eq. (12)**

Based on Eq. (11), now I show the procedure to derive the geometric discord for the thermal two-photon state of Eq. (5). The calculation is similar to that conducted for a family of high-dimensional states with high symmetry [6, 7], such as Werner states and pseudo pure states, where the white noise is a completely mixed state $I/d^{2}$. In contrast, here the background $\rho_{C}$ is a non-maximally mixed state, namely, $\rho_{C}\neq I/d^{2}$, due to the limited spiral spectrum of . Besides, the LG modes in our case are biorthorgonal in both and $p$ indices. This situation makes the calculation a little more complicated. We start with Eq. (5), and consider the index ranging from $-L$ to $+L$ while $p$ from $0$ to $P$, totally involving $(2L+1)(P+1)$ dimension. The pure entangled state contained in Eq. (7) is just the high-dimensional OAM entangled one. Following a similar procedure in Ref. [7], we assume the rank-1 POVM $\{|\eta_{jk}^{B}\rangle\langle\eta_{jk}^{B}|\}$ is performed on the photons in path *B* with , then by combining Eq. (5), we immediately know that the photon in path A will take the following state,

(S17)

According to Eq. (11), a key step is to calculate $\sum_{jk} \text{tr\{(}\rho_{jk}^{A})^{2}\}$. To this end, we first find that,

(S18)

As well known, majorization result says that since here$p_{jk}=\text{tr}\left\{_{B}\langle\eta_{jk}|\left.\Psi\right\rangle_{AB}\left\langle\Psi\right.|\eta_{jk}\rangle_{B}\right\}$ are the measurement statistics on the state $\text{tr}_{A}\left\{|\Psi\rangle_{AB}\left.\langle\Psi|\right\rangle\right\}$ whose eigenvalues are $P_{jk}^{2}$ [7, 8]. Along this line, we also have and . Consequently, we obtain form Eq. (S18) that,

(S19)

Besides, we also need to know $\text{tr\{}\rho^{2}\}$ in Eq. (11). A straightforward calculation based on Eq. (5) gives that,

. (S20)

By combining Eqs. (S19) and (S20) and taking the normalization condition, , into account, we finally obtain the geometric discord of Eq. (12) for the thermal two-photon state of Eq. (5).

**Reference**

1. L. Allen, M. J. Padgett, M. Babiker, "The Orbital Angular Momentum of Light," *Progress in Optics* **39**, 291–372 (1999).
2. A. Zeilinger, "General properties of lossless beam splitters in interferometry," *Am. J. Phys.* **49**, 882 (1981).
3. J. Leach, B. Jack, J. Romero, M. Ritsch-Marte, R.W. Boyd, A. K. Jha, S. M. Barnett, S. Franke-Arnold, and M. J. Padgett, "Violation of a Bell inequality in two-dimensional orbital angular momentum state-spaces," *Opt. Express* **17**, 8287-8293 (2009).
4. W. Martienssen and E. Spiller, "Coherence and fluctuations in light beams," *Am. J. Phys.* **32**, 919 (1964).
5. M. Abramowitz and I. A. Stegun, *Handbook of Mathematical Functions* (New York, Dover, 1965).
6. S. Luo and S. Fu, "Geometric measure of quantum discord," *Phys. Rev. A* 82, 034302 (2010)
7. E. Chitambar, "Quantum correlations in high-dimensional states of high symmetry," *Phys. Rev. A* **86**, 032110 (2012)
8. M. A. Nielsen and I. L. Chuang, *Quantum Computation and Quantum Information* (Cambridge University Press, Cambridge, 2000).
